# Decomposition Kinetics of Solution and Lyophilized Powder of S‐Nitroso‐N‐Cetylcysteine

**DOI:** 10.1111/1750-3841.71005

**Published:** 2026-04-01

**Authors:** Bruna Fernandes Andrade, Lorrany Ramos do Carmo, Vanelle Maria da Silva, Robledo de Almeida Torres Filho, Paulo Rogério Fontes, Alcinéia de Lemos Souza Ramos, Eduardo Mendes Ramos

**Affiliations:** ^1^ Departamento de Ciência dos Alimentos, Escola de Ciências Agrárias de Lavras Universidade Federal de Lavras Lavras Minas Gerais Brazil; ^2^ Instituto de Ciências Exatas e Tecnológicas Universidade Federal de Viçosa Florestal Minas Gerais Brazil; ^3^ Departamento de Tecnologia de Alimentos Universidade Federal de Viçosa Viçosa Minas Gerais Brazil

**Keywords:** Concentration, lyophilization, Nitrosothiol, reaction kinetics, stabilization, temperature

## Abstract

Recently, S‐nitrosothiols (RSNO) have received increased attention in the food industry as replacements for nitrite salts in meat products. However, their industrial applications are limited because of their high instability in concentrated aqueous solutions. In this study, S‐nitroso‐N‐acetylcysteine solutions (NAC‐SNO_Sol_) of different concentrations (200, 300, and 400 mM) were stabilized, impregnated with salt, and freeze‐dried (NAC‐SNO_Pow_). The decomposition kinetics of the solutions at 4°C and the resulting powders at different storage temperatures (–18 to 30°C) were evaluated using zero‐, first‐, and second‐order ordinary differential equations. The NAC‐SNO decomposition followed a first‐order reaction in non‐stabilized solutions and a zero‐order reaction in stabilized solutions, with the half‐life (*t*_1/2_) increasing from 1.1 to 9.9 days and decreasing in more concentrated solutions. The decomposition of NAC‐SNO_Pow_ had a better fit for second‐order decay, with an average activation energy (*E_a_*) of 92 kJ/mol and a *Q_10_* of 4.0. The stability of the powders increased with concentration, with *t*_1/2_ values reaching up to 100 days at the highest concentration and refrigeration temperature (2°C). Stabilizing and obtaining NAC‐SNO_Pow_ at higher concentrations and refrigerated or frozen storage is an interesting optimization strategy for industrial use, as it presents good yield (approximately 79%), stability, and practical use.

**Practical Applications:**

Previous studies demonstrated the potential use of RSNO solutions to replace the nitrite additive in meat products, maintaining the desired characteristics and reducing the risks of carcinogenic compound formation. However, the high instability of these compounds in solution limits their practical use. This research introduced an innovative approach by developing a more stable solid form of NAC‐SNO_Sol_ and determining the best storage conditions for use as a food additive. This advancement is crucial for enabling the industrial application of RSNO, making them a more accessible and efficient alternative for nitrite replacement in meat products.

## 1 Introduction

RSNO, also known as thionitrites, are a class of compounds widely studied in biological systems due to their ability to carry (“donate”) nitric oxide (NO). These compounds occur naturally in mammals and function mainly as signaling agents for physiological and pathophysiological processes (Liu et al. 2018). In vivo, NO carriers are amino acids or peptides containing a thiol group (R‐SH), but they can also be synthesized for pharmaceutical use, acting as vasodilators or in the treatment of diseases and poisoning (Yang et al. 2021). Furthermore, RSNOs have been studied in agriculture to improve plant defense and growth (Seabra et al. 2014), and recently as a potential food additive to replace curing salts (nitrite/nitrate) in meat products (Andrade, do Carmo et al. 2024; Andrade, Guimarães et al. 2024; Shpaizer et al. 2021; Shpaizer et al. 2018).

Curing salts (sodium or potassium nitrite and nitrate) are versatile food additives used as preservatives in various meat products. In addition to ensuring microbiological safety and extending shelf life, nitrite plays an important role in the meat matrix by controlling oxidative reactions and conferring important sensory characteristics (pinkish‐red color, flavor, and aroma) to cured products (Shakil et al. 2022). However, the use of curing salts in meat products has been questioned because of the risks arising from the formation of nitroso compounds (NOCs), some of which are mutagenic, teratogenic, and carcinogenic, such as N‐nitrosamines (IARC 2018), which are formed by N‐nitrosation reactions with secondary amines during product processing and stomach digestion (De Mey et al. 2017).

The total replacement of nitrite salts in meat products is an important and challenging current concern, as reformulated product needs to maintain sensory, microbiological, and stability characteristics during their shelf life compared to conventional products (Guimarães et al. 2022). Therefore, the proposal to replace nitrite with RSNOs is interesting because they share the same mechanism of action, specifically the generation of NO (Andrade, Guimarães et al. 2024). During the meat curing process, NO binds to the heme iron of the myoglobin (Mb) pigment, resulting in the formation of characteristic pigments in raw cured products (nitrosomyoglobin), with a pinkish‐red color, and in cooked products (nitrosohemochrome) with a pink color (Honikel 2008). The binding of NO to iron is also responsible for slowing oxidative processes by inhibiting its action as a catalyst, sequestering reactive oxygen species, and inhibiting the onset of lipid oxidation (Jo et al. 2020). Finally, the NO formed also has a bactericidal action by complexing with the iron of the metabolic enzymes of some microorganisms, such as *Clostridium botulinum*, and/or by forming the oxidant peroxynitrite through reactions with superoxide, which has a potent antimicrobial effect (Majou and Christieans 2018). According to Bryan et al. (2012), the direct delivery of NO reduces side reactions during curing, for example, with secondary amines, favoring S‐nitrosylation over N‐nitrosylation, thus reducing or inhibiting the formation of N‐nitrosamines.

The addition of RSNOs to meat products provides instrumental color and oxidative protection (Andrade, Guimarães, et al. 2024; Kanner and Juven, 1980), sensory characteristics (Andrade, do Carmo, et al. 2024), and anticlostridial activity (Shpaizer et al. 2021) similar to those of nitrite, but with a reduced tendency for N‐nitrosamine formation (Shpaizer et al. 2018). However, to date, studies on the application of RSNOs in the preparation of meat products have been restricted to the preparation and immediate use of solutions due to their spontaneous decomposition in aqueous media, which is a significant limitation to their storage and use as ingredients in the food industry.

RSNOs can be synthesized via a rapid and simple S‐nitrosation reaction between thiols and sodium nitrite (NaNO_2_)in an acidic medium. Generally, tertiary and secondary RSNOs are identified by green and red colors, respectively, and can be quantified using spectrophotometry at specific wavelengths (Williams 1985). However, the stability of RSNO solutions is influenced by factors such as concentration, pH, temperature, light, presence of metal ions (such as copper), and RSNO structure (secondary or tertiary), in addition to their spatial conformation (syn and anti) (de Souza et al. 2019; Meyer et al. 2016). The common RSNOs studied in the pharmaceutical field include S‐nitroso‐glutathione (Glu‐SNO), S‐nitroso‐L‐cysteine (Cys‐SNO), and S‐nitroso‐N‐acetyl‐L‐cysteine (NAC‐SNO), which are obtained from the S‐nitrosation of L‐cysteine, glutathione, and N‐acetyl‐L‐cysteine, respectively (Mathews and Kerr 1993). In studies with meat products, the NAC‐SNO has demonstrated superior technological performance when compared to other RSNOs (Andrade, Guimarães et al. 2024; Kanner and Juven 1980).

In this context, because of the instability of RSNO aqueous solutions, it is necessary to stabilize them to increase their shelf life and enable their use in the food industry. Kinetic studies on the stability of RSNO solutions have been conducted for pharmacological applications (de Souza et al. 2019), but these solutions are prepared at concentrations (<50 mM) that are unsuitable for meat product applications. Furthermore, the development of powdered RSNOs is important for industrial applications because they offer several advantages over other solutions, such as reduced packaging weight, greater stability and shelf life, and easier storage, handling, and transportation. Therefore, this study aimed to evaluate the kinetic degradation of NAC‐SNO solutions and powders obtained by solution stabilization and lyophilization during storage at different temperatures. The kinetic decomposition models can be used as a reference for the storage and preservation of this new curing agent, thereby expanding the prospects of its use as an ingredient in the food industry.

## 2 Materials and Methods

### 2.1 Synthesis and Decomposition Kinetics of NAC‐SNO Solutions

**FIGURE 1:**
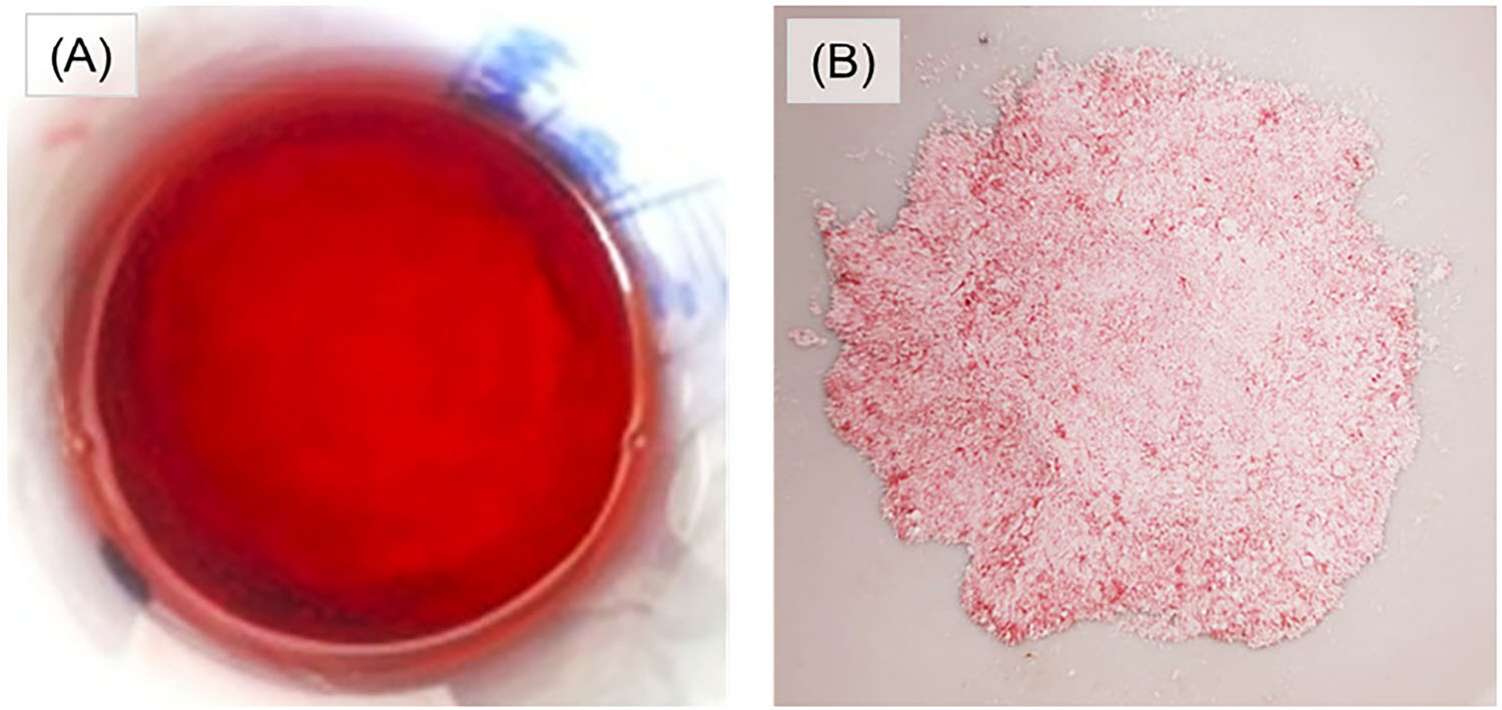
Photographs of S‐nitroso‐N‐acetylcysteine (NAC‐SNO; 300 mM) in (a) aqueous solution; and (b) powder (before macerating in porcelain pestler) forms.

NAC‐SNO was synthesized through an equimolar S‐nitrosation reaction between thiol, N‐acetyl‐L‐cysteine (NAC; Sigma‐Aldrich, St. Louis, MO, USA), and NaNO_2_ in an acidic medium, as described by Andrade, Guimarães et al. (2024). Three solutions of different concentrations were prepared: S1 = 200 mM, S2 = 300 mM, and S3 = 400 mM. NAC was diluted in an acidic solution (0.1 M HCl) and added to an aqueous solution of NaNO_2_ (in a ratio of 20:1) to obtain a mixture containing equimolar amounts of NAC and nitrite. The mixture was stirred at 30°C for 15 min, protected from light in amber glass vials, to allow the S‐nitrosylation reaction. The resulting solution (Figure 1A) was stabilized by neutralization with 1 M sodium hydroxide (NaOH) and adding phosphate salts to obtain a buffer (0.09 M; pH 7.0) solution and 0.1% ethylenediaminetetraacetic acid (EDTA) to chelate with possible metals present (de Souza et al. 2019).

The aqueous solutions (NAC‐SNO_Sol_; *n* = 3), stabilized (S1, S2, and S3) and non‐stabilized (SN; 300 mM), were portioned into 5‐mL amber glass screw‐cap vials which provided protection against light, and kept under controlled conditions in a climate chamber (EL202; Eletrolab Indústria e Comércio Ltda., São Paulo, SP, Brazil) at 4°C. The storage conditions were rigorously standardized to ensure the reliability of the results and to guarantee that any observed effects on NAC‐SNO decomposition were solely attributable to the controlled experimental variables. Temperature, light exposure, and the internal atmosphere of the vials were kept uniform in order to prevent variations in oxygen availability and the potential loss of NO‐related volatile species. It is well established that, under less controlled conditions, such fluctuations could influence the decomposition kinetics of NAC‐SNO and, in principle, affect data quality. Therefore, the vials remained tightly sealed throughout the entire storage period and were opened only briefly at the time of each sampling.

For the quantification of NAC‐SNO, the solutions were collected over a period of 30 days of storage or until the solutions reached a concentration of 1.0 mM. Sampling was conducted initially daily and later adjusted based on the observed decomposition rate. The amber vial was opened at each analysis time to collect a standardized aliquot (initially 10 µL) with a volumetric pipette. The concentrations of the solutions were quantified by reading the absorbance (Genesys 10 UV; Thermo Fisher Scientific Inc., Waltham, MA, USA) at 330 nm and considering a molar extinction coefficient (ε) of 727 M^−1^ cm^−1^ (Mathews and Kerr 1993). When necessary, the samples were diluted with distilled water to ensure that the absorbance readings remained within the reliable operating range of the spectrophotometer (0.200–0.800), where the instrument provides greater measurement accuracy and minimizes deviations

### 2.2 Synthesis, Freeze‐Drying, and Decomposition Kinetics of NAC‐SNO Powder

To obtain the NAC‐SNO powder (NAC‐SNO_Pow_; *n* = 3), stabilized solutions (S1, S2, and S3) were synthesized as previously described and added to sodium chloride salt (NaCl) in a ratio of 2:1 (v/m). The mixtures were frozen in an ultra‐freezer (Revco Dfx; Thermo Fisher Scientific Inc., Asheville, North Carolina, USA) at ‐70°C and lyophilized (Liobras‐L101; São Carlos, SP, Brazil) protected from light (bottles wrapped with aluminum foil). The average lyophilization time of the samples was 72 h, which resulted in powders with slightly pinkish hue (Figure 1B).

The lyophilized samples (P1, P2, and P3) were macerated in a mortar and pestle, stored in amber Eppendorf tubes, and randomly distributed in climatic chambers (Eletrolab Indústria e Comércio Ltda, São Paulo, SP, Brazil) or in a vertical freezer (Electrolux Cycle Defrost; Electrolux Brazil, Curitiba, PR, Brazil) for storage at temperatures of –18, 2, 7, 15 and 30°C. Quantification of NAC‐SNO in the powder was performed over 50 days of storage or until the concentration reached 1.0 mg/g. Sampling was initially conducted daily and later extended when decomposition slowed. An Eppendorf was opened for each analysis time, and NAC‐SNO_Pow_ (approximately 100 mg) was solubilized in a known volume of distilled water for its spectrophotometric quantification (ʎ = 330 nm; ε = 727 M^−1^ cm^−1^).

### 2.3 Statistical Modeling

The decomposition kinetic data of NAC‐SNO_Sol_, stabilized (S1, S2, and S3) and unstabilized (SN), and NAC‐SNO_Pow_ (P1, P2, and P3) were evaluated by fitting them to zero‐ (*k_0_*_;_ Equation 1), first‐ (*k_1_*_;_ Equation 2), and second‐order (*k_2_*_;_ Equation 3) reaction models. Reaction rate constants were calculated using linear regression.

(1) $$A=A_{0}-k_{0}t$$

(2) $$\ln\left(\frac{A}{A_{0}}\right)=-k_{1}t$$

(3) $$\left(\frac{1}{A}\right)=\left(\frac{1}{A_{0}}\right)+k_{2}t$$

where *A* is the concentration of the substance after time *t*, *A_0_* is the initial concentration, and *k_1_, k_2,_* and *k_3_* is the reaction rate constant.

The model and constant *k_1, 2,3_* best suited to the experimental data were selected using the coefficient of determination (*R^2^*) and root mean square error (RMSE; Equation 4). Using the best‐fitting model, the *t*_1/2_ was calculated as the time at which the NAC‐SNO concentration was reduced by 50% relative to the initial concentration. For NAC‐SNOP_Sol_, data extrapolation was performed to calculate a t_1/2_ greater than the evaluated storage time (50 days).

(4) $$\mathrm{RMSE}=\sqrt{\frac{1}{N}\sum_{i}^{N}{\left(y_{\exp,i}-y_{\mathrm{pred},i}\right)}^{2}}$$

where *y*_exp,*i*_ is the experimental value, *y*_*pre,i*_ is the predicted value for each linearized model, and *N* is the total number of data points. To evaluate the effect of temperature on the rate constant (*k*) of the NAC‐SNO_Pow_ degradation reaction, the activation energy (*E_a_*) required for the thermal cleavage of the single bond (S─NO) was determined by linear regression using the linearized Arrhenius equation (Equation 5).

(5) $$\ln\left(k\right)=-\frac{E_{a}}{R}\left(\frac{1}{T}\;-\;\frac{1}{T_{0}}\right)+ln\left(k_{pre}\right)$$

where *E_a_* is the activation energy (kJ/mol) and *R* is the universal gas constant (8.314 × 10–3 kJ/mol·K), *T* is the final absolute temperature (K), *T_0_* is the absolute reference temperature (K), *k* is the reaction rate constant, and *k_pre_* is the pre‐exponential factor.

The temperature acceleration factor (*Q_10_*), which corresponds to the multiplication or division value of the degradation rate when the temperature increases or decreases significantly by ten degrees, was determined by Equation 6.

(6) $$Q_{10}=10^{\left(\frac{E_{a}}{1,93\;\times \;T^{2}}\right)}$$

where *E_a_* is the activation energy (kJ/mol) and *T* is the average temperature (K).

All statistical analyses were performed using SAS System for Windows software, version 9.0 (SAS Institute Inc., Cary, NC). The means and standard deviations of the kinetic parameters (*k*, *t*_1/2_, *E_a_*, and *Q_10_*) were calculated from three independent measurements (repetitions) of the NAC‐SNO decomposition curves.

## 3 Results and Discussion

### 3.1 Kinetics of the Decomposition of NAC‐SNO Solutions

The decomposition of the NAC‐SNO solutions was studied by monitoring their concentrations in relation to the initial concentration over time. NAC‐SNO_Sol_ presented a better fit of the decomposition data (higher *R^2^* and smaller RMSE) to the first‐order reaction (k_1_) kinetic model for the SN solution and the zero‐order reaction kinetic (*k_0_*) model for the stabilized (S1, S2, and S3) solutions (Table 1). The reaction order mathematically indicates how the reaction rate is affected by the concentration of one or more reactants. The change in the kinetic behavior between the stabilized solutions and SN suggests that stabilization could alter the decomposition mechanism, reducing the direct dependence on concentration.

**TABLE 1:** Kinetic parameters of the decomposition of SN and stabilized^a^ (S1, S2, and S3) solutions of NAC‐SNO stored at 4°C protected from light.

Model	Solution	*k*	*R^2^*	RSME	*t*_1/2_ (days)
Zero order	SN	9.9051	0.88	10.05	—
	S1	1.0562	0.94	1.68	17.0
	S2	2.7667	0.94	0.38	9.9
	S3	9.4115	0.95	0.12	5.4
First order	SN	0.6239	0.94	0.21	1.1
	S1	0.0763	0.79	3.68	—
	S2	0.1677	0.91	0.31	—
	S3	0.3235	0.73	0.14	—
Second order	SN	0.0970	0.64	0.16	—
	S1	0.0106	0.41	6.12	—
	S2	0.0253	0.60	0.46	—
	S3	0.0526	0.42	0.16	—

*Note*: SN = 300 mM; S1 = 200 mM; S2 = 300 mM; S3 = 400 mM; *k =* reaction rate constant of zero (*k_0_*; mM/day), first (*k_1_*; /day) and second (*k_2_*; /mM×day) order; *R^2^* = coefficient of determination; RMSE = root mean square error; and *t*_1/2_ = half‐life.

^a^ Stabilized by neutralization with 1 M NaOH solution and addition of phosphate salts (0.09 M; pH 7.0) and 0.1% EDTA.

**FIGURE 2:**
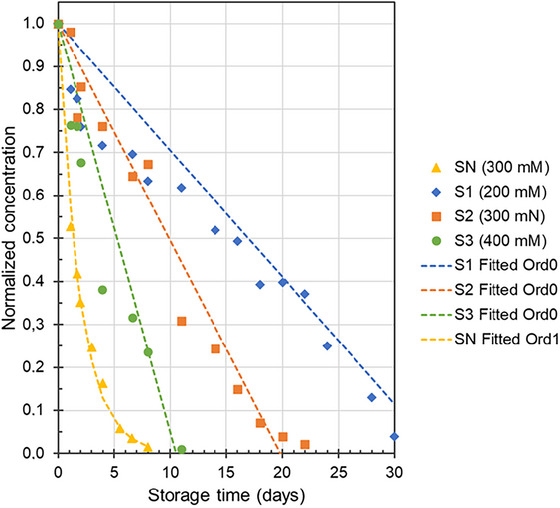
Zero‐ (Ord0) and first‐order (Ord1) kinetics curves of the decomposition of SN and stabilized (S1, S2, and S3) NAC‐SNO_Sol_ during storage (at 4°C) protected from light.

The NAC‐SNO_Sol_ concentration–storage time curves were fitted using zero‐order and first‐order decay equations (Figure 2), and the corresponding half‐lives (t_1/2_) were calculated. The zero‐order reaction rate (k_0_) of the stabilized solutions and the first‐order decomposition rate (*k_1_*) of the SN solution (SN; 300 mM) decreased by 3.58 and 3.72 times, respectively, when compared to the stabilized solution of the same concentration (S2). Thus, the t_1/2_ increased 9 times (from 1.1 to 9.9 days) during the stabilization process. When evaluating the effect of pH on 1 mM solutions of Glu‐SNO and NAC‐SNO, de Souza et al. (2019) found that the decomposition of RSNOs tends to be favored at pH values below 5.0 or above 9.0, with greater stability at pH close to neutral. Although this study evaluated extremely high concentrations, similar results were obtained, strongly suggesting the need to adjust the pH close to neutral after the S‐nitrosation reaction. In the SN solutions, the final pH values were close to 2.0, which was necessary for the synthesis process. During storage, RSNO solutions tend to become acidic due to decomposition into NO and the formation of nitric acid (HNO_2_). Thus, the observed stabilization can be attributed primarily to the adjustment of the pH to values near neutrality, a condition that significantly slows the decomposition rate of RSNOs. Complementarily, the presence of EDTA may have contributed to additional stabilization, since EDTA is a chelating agent for metal ions, such as Cu^2^^+^ and Hg^2^^+^, which are known to catalyze RSNO decomposition (Li et al. 2022). The combination of these factors promotes greater stability of NAC‐SNO during storage.

In the stabilized solutions, *k* increased with increasing NAC‐SNO concentration (Table 1). With respect to the *k_0_*, raising the NAC‐SNO concentration from 200 mM (S1) to 400 mM (S3) led to an 8.91‐fold increase in the reaction rate constant. Similar behavior was reported by de Souza et al. (2019), who observed that more dilute concentrations of stabilized Glu‐SNO and NAC‐SNO solutions (1 to 50 mM) were significantly more stable at 25°C than concentrated solutions. According to these authors, the initial concentration of RSNOs influences the mechanism and, consequently, the decomposition reaction rate, with the main decomposition pathway being the spontaneous homolytic cleavage of the S‐N bond at low initial concentrations. In more concentrated solutions, the preferred mechanism is autocatalysis, that is, the thiyl radical (R‐S) acts as a catalyst for the decomposition of other intact RSNOs, generating dimers (R‐S‐S‐R) and NO. Some studies in medicinal chemistry have suggested that increasing the viscosity of RSNO solutions (Shishido and de Oliveira 2000) or incorporating RSNOs into solid matrices (Seabra et al. 2004) can reduce the autocatalytic decomposition rate.

Although the concentrations evaluated in this study are much higher (4 to 8 times) than the maximum concentration (50 mM; k_1_ = 0.325/day) of NAC‐SNO monitored by de Souza et al. (2019), lower *k* values were observed for the stabilized solutions, which can be attributed to the addition of stabilizers associated with the lower storage temperature (4°C vs. 25°C). A significant increase in the decomposition rate of RSNOs was observed with increasing storage temperature owing to the increase in the thermal energy available in the system (Melvin et al. 2019). In addition to temperature, the decomposition process of RSNOs can be influenced by different mechanisms depending on conditions such as the specific chemical structure of the evaluated species, concentration, pH, presence of catalysts, light, and oxygen (Dorado et al. 2015).

### 3.2 Kinetics of Decomposition of NAC‐SNO Powder

Freeze‐drying of the stabilized solutions (NAC‐SNO_Sol_) impregnated with salt‐generated powders (NAC‐SNO_Pow_) containing 38.42 ± 0.98 mg (P1), 71.73 ± 0.99 mg (P2), and 109.49 ± 4.09 mg (P3) of NAC‐SNO/g. Thus, the decomposition of NAC‐SNO in solution during the process provided an average yield of approximately 79%.

The best kinetic model fit (highest *R^2^* and lowest RMSE) observed for NAC‐SNO_Pow_ was the second‐order reaction (Table 2), with lower *k_2_* values found at the lowest temperature evaluated (–18°C). As expected, an increase in *k_2_* values was observed with increasing storage temperature (up to 30°C), i.e., there is an increase in stability with decreasing storage temperature. Similar results were reported for RSNO solutions (de Souza et al. 2019; Melvin et al. 2019). Chemical reactions can be understood by the collision theory, which assumes that molecules must collide effectively with each other for a reaction to occur. The efficiency of collisions can be affected by the frequency of collisions, proper orientation between molecules, and energy at the time of collision. Therefore, increasing the temperature increases the kinetic energy of the molecules and consequently increases the probability of effective collisions occurring (Koga et al. 2023). To the best of our knowledge, a kinetic study on the stability of RSNO powders has not yet been reported in the literature.

**TABLE 2:** Kinetic parameters of the decomposition of stabilized^1^ and salt‐impregnated NAC‐SNO at different concentrations (P1, P2, and P3), stored at various temperatures (T) protected from light.

—	—	P1 (38 mg SNAC/g)			P2 (72 mg SNAC/g)			P3 (110 mg SNAC/g)		
Model	T (°C)	*k*	*R^2^*	RMSE	*k*	*R^2^*	RMSE	*k*	*R^2^*	RMSE
Zero order	−18	0.1755	0.51	2.76	0.2633	0.62	3.22	0.2031	0.43	3.67
	2	0.3460	0.69	4.02	0.6125	0.84	5.68	0.6623	0.78	6.48
	7	0.5600	0.63	4.45	1.5779	0.92	4.76	0.9462	0.57	11.72
	15	0.4388	0.49	4.55	0.8492	0.49	9.05	1.5648	0.76	9.89
	30	2.8107	0.88	0.56	2.7259	0.85	0.87	10.2283	0.90	3.19
First order	−18	0.0058	0.56	0.08	0.0043	0.64	0.05	0.0020	0.43	0.04
	2	0.0266	0.77	0.25	0.0139	0.89	0.11	0.0079	0.80	0.07
	7	0.0622	0.88	0.23	0.0689	0.94	0.18	0.0138	0.61	0.15
	15	0.0726	0.75	0.42	0.0447	0.69	0.33	0.0270	0.87	0.13
	30	0.8141	0.96	0.07	0.3189	0.83	0.10	0.4851	0.95	0.09
Second order	−18	0.0002	0.61	<0.01	0.0001	0.66	<0.01	0.0000	0.43	<0.01
	2	0.0024	0.79	0.02	0.0003	0.92	<0.01	0.0001	0.81	<0.01
	7	0.0097	0.97	0.02	0.0051	0.80	0.03	0.0002	0.64	<0.01
	15	0.0184	0.93	0.05	0.0030	0.86	0.01	0.0005	0.94	<0.01
	30	0.2839	0.98	0.02	0.0404	0.80	0.01	0.0262	0.95	0.01

Abbreviations: *k =* reaction rate constant of zero (*k_0_*; mM/day), first (*k_1_*; /day) and second (*k_2_*; /mM×day) order; R^2^ = coefficient of determination; and RMSE = root mean square error.

*Note*: ^1^Stabilized by neutralization with 1 M NaOH solution and addition of phosphate salts (0.09 M; pH 7.0) and 0.1% EDTA.

**FIGURE 3:**
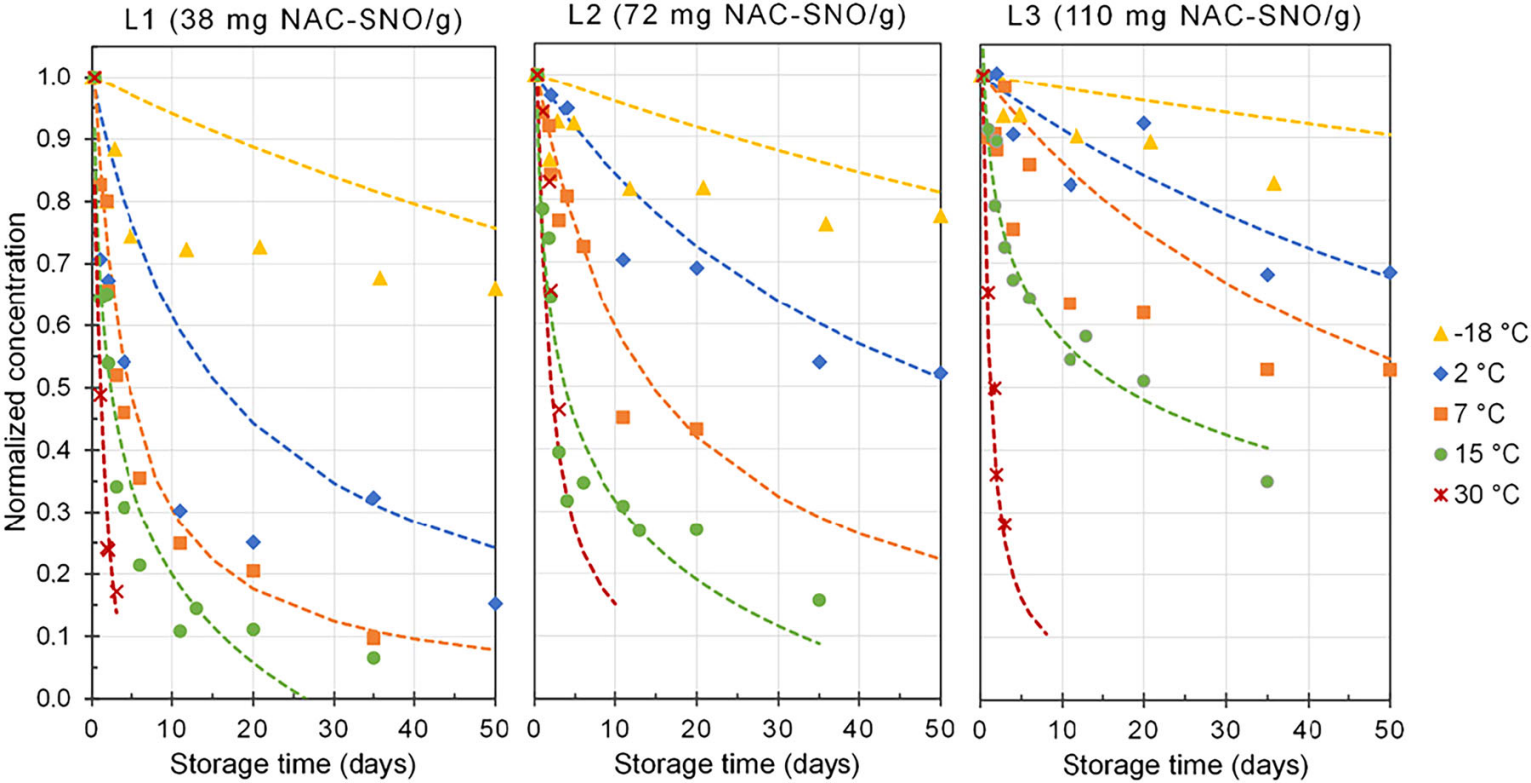
Second‐order kinetic curves of the decomposition of NAC‐SNO_Pow_ at different concentrations (P1, P2, and P3) during storage (protected from light) at different temperatures.

The NAC‐SNO_Pow_ concentration–storage time curves were fitted using second‐order decay equations (Figure 3). Contrary to what was observed for the solutions, there was an increase in the *k_1_* and *k_2_* values with decreasing concentrations of NAC‐SNO impregnated into the salt, regardless of the storage temperature (Table 2). The reactions between reactants in different physical states are naturally different. In general, reactions in solids are expected to be slower than those in solutions because the molecules have lower mobility and kinetic energy. In addition, solids have a smaller contact surface available for reaction; therefore, the reaction is limited to their surface. In solution, the presence of the solvent tends to facilitate collisions and increase the reaction rate because the reactants can interact throughout the entire liquid. Furthermore, the adsorption effect of NAC‐SNO_Sol_ on NaCl may also have contributed positively to stability, reducing the availability of NAC‐SNO for the reaction. According to Dorado et al. (2015), the decomposition process of RSNOs can occur via different mechanisms depending on the chemical structure, concentration of the species evaluated, temperature, pH, and the presence of catalysts such as light, oxygen, and metals. In addition, some ionic species in the solution can interfere with its stability (Demaster et al. 1997).

Although drying can favor the stability of the target compound by controlling chemical interactions, it also increases exposure to oxygen (not removed during storage in this experiment); therefore, decomposition of NAC‐SNO_Pow_ occurs predominantly via oxidation mediated by molecular oxygen. According to Zhang et al. (2017), primary and secondary RSNOs have low stability under aerobic conditions, with the extent of decomposition dependent on the oxygen concentration. According to these authors, the decomposition mechanism can be understood as the homolytic cleavage of the S‐NO bond, followed by the reaction of the NO radical with oxygen, forming nitrous anhydride (N_2_O_3_), a species with high oxidizing power responsible for the decomposition of RSNO. In the presence of N_2_O_3_, the chloride ion (Cl^−^) can form an intermediate nitrosated compound (ClNO), which can be hydrolyzed to nitrite (NO_2_^−^) or nitrosate thiol groups (Demaster et al. 1997), propagating the oxidation of NAC‐SNO.

Furthermore, because each Eppendorf tube was opened only once for analysis, the amount of oxygen was probably the same across samples. Thus, at higher concentrations of NAC‐SNO_Sol_, oxygen was the limiting reagent in the oxidation reaction during compound degradation. This, combined with the lower exposure of the more concentrated NAC‐SNO_Pow_, could result in their lower decomposition by oxidation, as observed by their lower *k_1_* values, and therefore, their greater stability. These findings suggest that using an inert atmosphere (such as vacuum or nitrogen) during the stabilization and storage of NAC‐SNO_Pow_ may be a strategy to further reduce their decomposition and increase their stability.

Lower decomposition of NAC‐SNO was observed in more concentrated powders, resulting in a higher *t*_1/2_ (Table 3). In general, considering a second‐order reaction and the extrapolation of the evaluated time interval (50 days), the *t*_1/2_ values of NAC‐SNO_Pow_ were lower than 20 days at storage temperatures above refrigeration (15 and 30°C), limiting their suitability for industrial application; with the decrease to refrigeration temperatures, the t_1/2_ of the powders increased to approximately 53 (P2 at 2°C and P3 at 7°C) and 101 (P3 at 2°C) days; at freezing temperature (–18°C), the estimated t_1/2_ were higher than 142 days, with the longest time observed at the highest concentration (P3) of NAC‐SNO_Pow_.

**TABLE 3:** Means and standard deviation^1^ of the *t*_1/2_, *Ea*, and temperature acceleration factor (*Q_10_*) estimated (second‐order model) for the decomposition of NAC‐SNO_Pow_ at different concentrations (P1, P2, and P3) and stored at various T protected from light.

T (°C)	P1 (38 mg NAC‐SNO/g)			P2 (72 mg NAC‐SNO/g)			P3 (110 mg NAC‐SNO/g)		
	*t*_1/2_ (days)	*E_A_* (kJ/mol)	Q_10_	*t*_1/2_ (days)	*E_A_* (kJ/mol)	*Q_10_*	*t*_1/2_ (days)	*E_A_* (kJ/mol)	*Q_1_*_0_
−18	142.84 ± 11.06	96.1 ± 2.1	4.3 ± 0.1	265.69 ± 47.6	83.7 ± 4.7	3.6 ± 0.3	520.57 ± 218.3	91.0 ± 7.0	4.0 ± 0.4
2	11.54 ± 1.34	(*R^2^* = 0.96)	—	56.75 ± 10.37	(*R^2^* = 0.88)	—	101.67 ± 10.63	(*R^2^* = 0.85)	—
7	2.88 ± 0.21	—	—	3.62 ± 0.49	—	—	49 ± 14.61	—	—
15	1.54 ± 0.20	—	—	6.37 ± 1.43	—	—	19.2 ± 0.86	—	—
30	0.1 ± 0.01	—	—	0.57 ± 0.36	—	—	0.38 ± 0.09	—	—

*Note*: ^1^Calculated by extrapolating data when t_1/2_ was greater than the 50‐day storage time evaluated.

The (*E_a_* is the minimum energy required for the reaction to occur and was calculated with values of 84 to 96 kJ/mol (13 to 17 kcal/mol) for the second‐order model in the temperature range evaluated (–18 to 30°C). These values were lower, but close to those found for solutions with concentrations equal to or lower than 50 mM of NAC‐SNO (22 kcal/mol), as reported by de Souza et al. (2019). According to Stamler and Toone (2002), RSNOs have homolytic bond dissociation energies in the range of 20 to 32 kcal/mol under conditions in which light and oxygen are excluded. Therefore, lower values may be associated with the conditions of this experiment, such as the highest concentration evaluated and the presence of oxygen, albeit at low concentrations.

The activation energies obtained for the different NAC‐SNO_Pow_ concentrations did not show a direct relationship with the concentration. There was only a variation of approximately 10% between the extreme values obtained, with an average value of 92.3 kJ/mol for the second‐order reaction. Like energy activation, the Q_10_ value varied slightly between NAC‐SNOPs, with an average value of 4.0. This high Q_10_ value demonstrates the importance of reducing the temperature to reduce the degradation rate of NAC‐SNO_Pow_ and, therefore, to obtain greater stability.

## 4 Conclusion

Stabilization of the NAC‐SNO solutions induced a nine‐fold increase in the half‐life compared to that of the SN solutions, which increased 100‐fold upon lyophilization and powder salt coprecipitation. Furthermore, the effect of concentration on the decomposition of NAC‐SNO depended on its physical state, being favored at higher concentrations in solution and unfavored in the powder form. These results support the use of stabilized NAC‐SNO powders as a promising strategy for industrial applications, offering good yield, stability, and practicality of use. Furthermore, the use of higher concentrations of NAC‐SNO for salt coprecipitation and frozen storage has been suggested to achieve greater stability.

## 5 Future Directions

The development of RSNOs in solid form represents a significant advancement for their industrial application, as it overcomes limitations associated with the use of diluted solutions, which would require large storage volumes, appropriate infrastructure, and the handling of acidic solutions within the food industry. To consolidate this innovation, however, it is essential to evaluate its applicability across different categories of meat products. This is due to the wide diversity of formulations, which vary in ingredients, additives, fat content, and pigments that may interact differently with RSNOs, in addition to the influence of technological processes such as drying, smoking, and fermentation, which can alter their stability and effectiveness.

It is also necessary to investigate the differences between restructured and whole‐muscle products, in which curing brines are used, since it is not yet fully understood how the diffusion of RSNO‐containing solutions would occur or how effective this process would be. Another critical point is to clarify their role in microbial control, particularly against Clostridium botulinum and other pathogens, as the mechanisms of action remain unclear and the ideal concentration required to ensure product safety has not yet been established.

From a sensory perspective, it is indispensable to determine whether RSNOs leave residual flavors, enhance desirable attributes, or compromise the sensory stability of products. Finally, research should prioritize the investigation of nitrosamines derived from these compounds, since, as nitrosating agents, RSNOs may generate new species not yet described in the literature, whose toxicological risks may differ — and even exceed in severity — those associated with traditionally formed nitrosamines.

## Author Contributions

**Bruna Fernandes Andrade**: methodology, investigation, writing – original draft. **Lorrany Ramos do Carmo**: investigation. **Vanelle Maria da Silva**: validation, formal analysis, writing – review and editing. **Robledo de Almeida Torres Filho**: validation, formal analysis, writing – review and editing. **Paulo Rogério Fontes**: writing – review and editing. **Alcinéia de Lemos Souza Ramos**: methodology, supervision, writing – review and editing. **Eduardo Mendes Ramos**: conceptualization, methodology, supervision, resources, project administration.

## Conflicts of Interest

The authors declare no conflicts of interest.
